# From Scores to Scholarship: Evolving Metrics in Vascular Surgery Residency Admissions

**DOI:** 10.1177/15385744251410008

**Published:** 2025-12-23

**Authors:** Ismail Zazay, James R. Burmeister, Jose I. Ortiz De Elguea-Lizarraga, Lily Cormier, Annie Cherner, Mitchell Cox

**Affiliations:** 1 John Sealy School of Medicine, 12338 University of Texas Medical Branch, Galveston, TX, USA; 2 159878 Oakland University William Beaumont School of Medicine, Rochester, MI, USA; 3 Department of Vascular Surgery, 12338 University of Texas Medical Branch, Galveston, TX, USA

**Keywords:** research, vascular surgery, USMLE Step 1, medical education, residency match

## Abstract

**Background:**

In 2022, the United States Medical Licensing Examination (USMLE) Step 1 transitioned to a pass/fail format, removing a long-standing objective measure from residency applications. This shift led applicants and/or residency program directors to place increased emphasis on alternative metrics such as research output (RO), though how much programs value this remains unclear. This study explores RO trends among vascular surgery applicants from 2014-2024 and compares them by applicant type and across other competitive surgical specialties.

**Methods:**

NRMP Charting Outcomes in the Match reports (2014-2024) were reviewed. The mean number of abstracts, presentations, and peer-reviewed publications (RO) was collected for matched and unmatched U.S. MD seniors applying to vascular surgery. RO data for Doctor of Osteopathic Medicine (DO) and non-U.S. international medical graduate (IMG) applicants were also reviewed for completeness.

**Results:**

RO among matched U.S. MD vascular surgery applicants rose by 54.7%, from 7.0 in 2014 to 12.8 in 2024. The most marked increase occurred between 2018 and 2022 (8.3 to 12.4; +67%), with growth plateauing thereafter after 2022. In contrast, RO among matched DO and IMG applicants declined: DO applicants dropped from 21.3 in 2022 to 9.0 in 2024, and IMG applicants from 60.3 in 2020 to 38.3 in 2024. However, 2024 sample sizes were small, 137 matched U.S. MDs vs only 3 DOs and 6 IMGs, limiting direct comparisons.

**Conclusion:**

While RO among vascular surgery applicants surged after Step 1 became pass/fail, the recent plateau suggests a transient response rather than ongoing growth, differing from trends in other surgical subspecialties. These findings raise concerns about equity and added pressure on underrepresented applicants. Further research is needed to clarify RO’s actual role in residency selection and inform fairer evaluation practices.

## Introduction

Vascular surgery, though numerically smaller than general surgery, remains highly competitive, offering approximately 150 integrated and independent residency positions annually. Historically, United States Medical Licensing Examination (USMLE) Step 1 numeric scores were pivotal in applicant evaluation. The 2022 transition of Step 1 to pass/fail grading removed a key standardized metric, potentially prompting applicants to increase their research output (RO), defined by the National Resident Matching Program (NRMP) as the sum of abstracts, presentations, and peer-reviewed publications, to remain competitive. However, it remains uncertain whether this response was driven by applicant perception or by actual shifts in program director priorities.^1,2^

This study examines changes in RO among vascular surgery applicants using NRMP data from 2014 to 2024. Trends are compared across different applicant types, including U.S. Medical Doctors (MDs), Doctors of Osteopathic Medicine (DOs), and non-US international medical graduates (IMGs).

## Materials and Methods

Publicly available NRMP *Charting Outcomes in the Match* reports from 2014 to 2024 were reviewed. Mean numbers of RO were extracted for matched and unmatched U.S. MD seniors applying to vascular surgery. RO data were also reviewed for DO and IMG applicants when available. Due to small sample sizes, especially among DO and IMG applicants, descriptive analysis was emphasized over statistical comparison. In 2024, there were 137 matched MD seniors, compared to only 3 DOs and 6 IMGs, which limits the generalizability of the findings for DO and IMG applicants.

## Results

**Figure 1.**
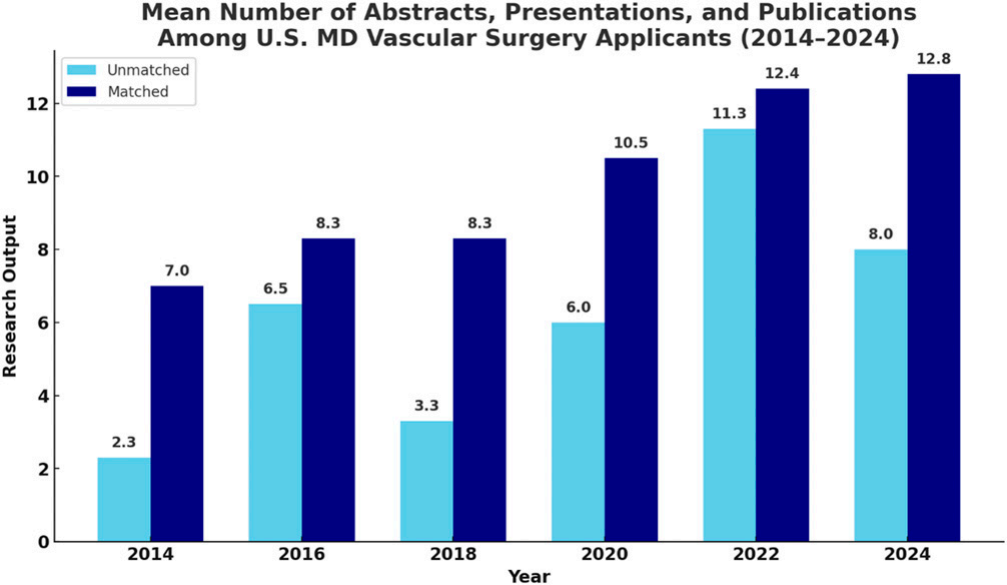
Mean number of abstracts, presentations, and peer-reviewed publications, defined as research output (RO) among U.S. MD Integrated Vascular Surgery residency applicants from 2014 to 2024, stratified by match status. Applicants who successfully matched had consistently higher mean RO across all years compared to unmatched applicants. Notably, RO increased for both groups over the decade, with the most substantial growth observed after 2020. These findings suggest a rising emphasis on scholarly activity in the vascular surgery match process

Between 2014 and 2024, RO among matched MD vascular surgery applicants increased by 54.7%, from 7.0 to 12.8. The steepest gains in RO occurred between 2018 and 2022, increasing from 8.3 to 12.4 (67%), coinciding with the rollout of Step 1 pass/fail scoring, implemented in January 2022 (Figure 1).^3^ After 2022, however, the rise in RO plateaued, increasing only modestly from 12.4 to 12.8.

**Figure 2.**
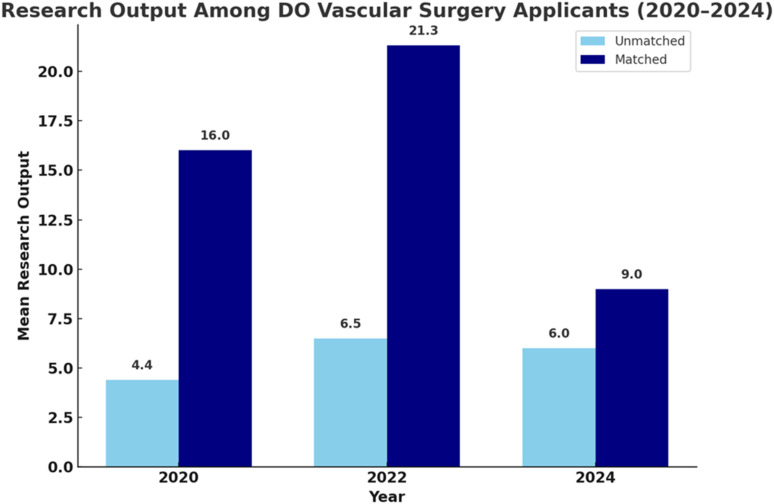
Mean research output (RO) among Doctor of Osteopathic Medicine (DO) applicants to Integrated Vascular Surgery residency programs from 2020 to 2024, stratified by match status. Matched applicants consistently demonstrated higher mean RO compared to their unmatched counterparts across all years. The greatest disparity was observed in 2022, suggesting that RO may be associated with match outcomes among DO candidates.

**Figure 3.**
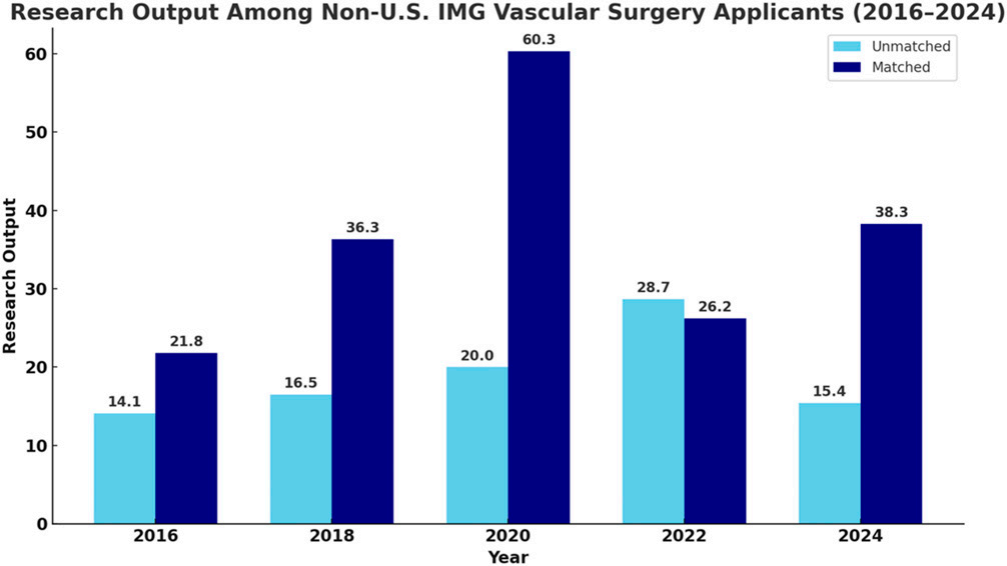
Mean research output (RO) among non–U.S. International Medical Graduate (IMG) applicants to Integrated Vascular Surgery residency programs from 2016 to 2024, stratified by match status. Matched IMG applicants generally demonstrated higher RO compared to unmatched applicants. A sharp peak in RO was observed in 2020 among matched applicants, with a mean of 60.3 peer-reviewed publications, abstracts, and presentations. Despite fluctuations over time, this trend highlights the potential role of RO in match outcomes of among IMG applicants

In contrast, RO among matched DO and IMG applicants declined in recent years. DO applicant RO decreased from 21.3 in 2022 to 9.0 in 2024, and IMG applicant RO dropped from 60.3 in 2020 to 38.3 in 2024 (Figures 2 and 3).^3^ These data should be interpreted cautiously, as sample sizes were small, only 3 DOs and 6 IMGs matched into vascular surgery in 2024, compared to 137 MDs.^3^ The limited representation makes broad generalizations difficult and may exaggerate year-to-year variations seen in DO and IMG applicants.

## Discussion

While RO among MD vascular surgery applicants increased following the Step 1 transition from numerical scoring to pass/fail, this trend appears to have plateaued after 2022, diverging from the continued acceleration observed in other competitive surgical subspecialties. For instance, otolaryngology matched applicants more than doubled their RO from 8.4 in 2016 to 17.2 in 2022.^4^ Plastic surgery applicants showed a steady rise from 19.1 in 2020 to 34.7 in 2024.^5^ Recent data from psychiatry show a similar trend: mean RO among matched applicants increased from 3.8 in 2014 to 7.5 in 2024, with the gap between matched and unmatched applicants widening substantially after Step 1 became pass/fail. This underscores that RO has become a key differentiator in the match process of multiple specialties, including those previously considered less research-driven.^6^

Despite these widespread increases in RO across other disciplines, vascular surgery appears to be an exception, showing early growth followed by a recent plateau. Understanding the factors behind this divergence is critical to contextualizing how the specialty’s structure and culture influence applicant research engagement.

Firstly, vascular surgery remains a small academic field with fewer integrated programs and limited research infrastructure compared to fields such as plastic surgery or otolaryngology. Many institutions lack a vascular-specific research pipeline or dedicated faculty mentorship, which may limit research opportunities for interested students.^4^

Second, although the clinical demands of vascular surgery are most evident during residency and fellowship, these characteristics are often well known to medical students and may shape their early perceptions of the field. The prospect of long hours, high-acuity cases, and demanding call schedules can discourage students from pursuing vascular-specific research or dedicating a research year to the specialty. Thus, even before entering residency, perceived workload may indirectly influence medical students’ engagement in vascular surgery scholarship.

In contrast to initial assumptions, DO and IMG RO has not increased in recent years, but declined. The minimal representation of DO (n = 3) and IMG (n = 6) applicants in 2024 limits the interpretability of their data. Furthermore, these groups may face systemic barriers, including fewer research resources, limited access to mentorship, and potential bias in the residency selection process. Dr. Saddawai-Konefka, Assistant Professor of Anaesthesia at Harvard Medical School, has mentioned how the chances of matching as an IMG or DO are lower.^7^

Beyond applicant type, the national surge in RO has raised additional concerns. For instance, “gift authorship”, a process in which individuals are listed on publications despite having made little or no meaningful contribution. Although collaboration is a cornerstone of contemporary research, distinguishing meaningful contributions from honorary authorship can be challenging, particularly within student-led research networks that span multiple institutions. This trend threatens the credibility of RO as a selection metric and reflects the pressure applicants face to meet inflated expectations.^8^

Current match trends suggest that both applicants and residency program directors may be prioritizing quantity over quality of research. While RO has become a key differentiator, less attention is paid to the nature of the work. For example, how does the type of article influence the value that residency program directors place on a publication? Are original research articles weighted more heavily than case reports? Additionally, how does authorship position, such as first author, second author, or senior author, affect the perceived importance of a publication? Future research should investigate how these factors shape program directors’ perceptions and whether publishing in a vascular-specific journal enhances an applicant’s competitiveness in the vascular surgery match.

A shift toward emphasizing quality over quantity must be accompanied by structural reforms. National efforts to support multicenter collaboration, remote-access research, and funding for under-resourced institutions would expand opportunities for underserved medical students. Programs must also recognize that RO is an imperfect proxy for applicant strength, and that an overreliance on scholarly output may crowd out more meaningful clinical and educational qualities (Figures 2 and 3).

## Conclusion

RO among vascular surgery applicants increased significantly from 2014 to 2024, with the sharpest gains occurring after the USMLE Step 1 scoring transition from a numerical score to pass/fail in 2022. However, unlike other competitive specialties, this rise did not continue at the same exponential pace in recent years; RO increased only modestly from 12.4 to 12.8 between 2022 and 2024.^3^ This plateau suggests a short-term applicant reaction rather than a sustained structural shift.

Meanwhile, RO among DO and IMG applicants has declined, and their outstandingly small sample sizes limit robust interpretation. The decrease in RO among these groups may be due to systemic bias and reduced access to research opportunities. More broadly, the continued pressure to increase RO raises concerns about equity, academic integrity, and applicant well-being, particularly for students from underserved programs and less research-intensive institutions.

Moving forward, a more nuanced approach to research evaluation is essential. Understanding the impact of article type, authorship position, journal specificity, and publication quality will help shift the emphasis away from raw numbers. Programs and policymakers should work toward a fairer, more transparent residency selection process that values quality over quantity and expands access to meaningful academic development for all applicants.
